# Opposing influences of *TAC1* and *LAZY1* on Lateral Shoot Orientation in Arabidopsis

**DOI:** 10.1038/s41598-020-62962-4

**Published:** 2020-04-08

**Authors:** Courtney A. Hollender, Joseph L. Hill, Jessica Waite, Chris Dardick

**Affiliations:** 1 Department of Horticulture, Michigan State University, East Lansing, MI 48824 USA; 2 USDA-ARS Appalachian Fruit Research Station, Kearneysville, WV 25430 USA; 3 Present Address: Washington State University Tree Fruit Research and Extension Center, Wenatchee, WA 98801 USA

**Keywords:** Plant development, Plant genetics, Plant molecular biology

## Abstract

*TAC1* and *LAZY1* are members of a gene family that regulates lateral shoot orientation in plants. *TAC1* promotes outward orientations in response to light, while *LAZY1* promotes upward shoot orientations in response to gravity via altered auxin transport. We performed genetic, molecular, and biochemical assays to investigate possible interactions between these genes. In Arabidopsis they were expressed in similar tissues and double mutants revealed the wide-angled *lazy1* branch phenotype, indicating it is epistatic to the *tac1* shoot phenotype. Surprisingly, the lack of *TAC1* did not influence gravitropic shoot curvature responses. Combined, these results suggest *TAC1* might negatively regulate *LAZY1* to promote outward shoot orientations. However, additional results revealed that *TAC1-* and *LAZY1* influence on shoot orientation is more complex than a simple direct negative regulatory pathway. Transcriptomes of Arabidopsis *tac1* and *lazy1* mutants compared to wild type under normal and gravistimulated conditions revealed few overlapping differentially expressed genes. Overexpression of each gene did not result in major branch angle differences. Shoot tip hormone levels were similar between *tac1*, *lazy1*, and Col, apart from exceptionally elevated levels of salicylic acid in *lazy1*. The data presented here provide a foundation for future study of *TAC1* and *LAZY1* regulation of shoot architecture.

## Introduction

Lateral organ orientation in both shoots and roots plays a key role in a plant’s interaction with the environment and its ability to access resources such as light and water. The IGT gene family members *TILLER ANGLE CONTROL 1* (*TAC1*) and the related set of *LAZY* genes are important regulators of lateral organ orientation^1,2^. These genes share four conserved amino acid regions or domains, and *LAZY* genes share an additional C-terminal domain^3,4^. *TAC1* generally occurs as a single copy gene, but many species possess multiple *LAZY* genes, with six identified in Arabidopsis^2–8^. While the *LAZY1* gene of *Arabidopsis thaliana* (Arabidopsis) contributes almost exclusively to shoot architecture, the remaining Arabidopsis *LAZY* genes primarily control root architecture^7,8^. However, GUS reporter activity and additive shoot phenotypes in plants with combinatorial *lazy1*, *lazy2*, and *lazy4* mutations suggest *LAZY2* and *LAZY4* also have a role in regulating shoot orientation^7,8^. *LAZY2*, 3, and 4 are also known as *DEEPER ROOTING* (*DRO3, 2, & 1*) and *NEGATIVE GRAVITROPIC RESPONSE OF ROOTS* (*NGR1, 3, & 2*), respectively, as they were separately identified as regulators of lateral root orientation^6,9–12^. Further, an alternate *LAZY* gene nomenclature denotes *LAZY3* as *LZY2* and *LAZY4* as *LZ3Y*^8^.

Lateral branches, tillers, leaves, and flower buds of plants with loss-of-function *tac1* mutations or reduced *TAC1* expression exhibit upright orientations^3,13–19^. In contrast, *lazy1* mutants have wider branch or tiller angles and *lazy4/dro1* mutants display prostrate lateral root orientations^4–9,20–24^. Plants with multiple *lazy/dro* mutations exhibit even wider lateral shoot/root angles and, in some cases, downward shoot growth and/or upward root growth^7,8,10,11^. Together, those findings demonstrate that *TAC1* promotes horizontal lateral organ orientations, while *LAZY* genes promote vertical orientations. Despite their sequence similarity and opposing roles in shoot architecture, functional relationships between *TAC1* and the *LAZY/DRO* genes have not been identified to date.

Currently, very little is known about *TAC1* function, but significant progress has been made uncovering *LAZY/DRO* functional mechanisms. *LAZY/DRO* family members regulate gravitropic responses in shoots and/or roots downstream of amyloplast sedimentation and upstream of the establishment of the asymmetric auxin hormone gradients generated by PIN auxin-efflux proteins^4,5,7,8,12,20,21,25,26^. *LAZY/DRO* genes are expressed in vasculature and gravity sensing tissues^4,7,8,12,21^. Single *lazy1* and multiple *lazy/dro* mutants have impaired, or in some cases reversed, gravitropism phenotypes, yet exhibit normal amyloplast development and sedimentation in response to gravistimulation^4,5,7,8,12,20,21,25,26^. *Oryza sativa* (rice) and *Zea mays* (maize) *lazy1* mutants have increased basipetal (root-ward) polar auxin transport and reduced lateral auxin transport, including gravity-induced transport in response to gravistimulation^5,20,21,26^. Additionally, *lazy1* maize showed decreased expression of *PIN1c*, and agravitropic Arabidopsis *lazy1;lazy2;lazy4* and *lazy2;lazy3;lazy4* roots exhibited reversed auxin gradients and polar PIN3-GFP localization upon gravistimulation^5,8,12^

An N-terminal transmembrane domain (TMD) and two C-terminal Nuclear Localization Signals (NLS) have been identified in LAZY1 and other LAZY proteins^4,27^. Yet, the importance or specific role of each domain may vary between species. Heterologously expressed GFP-tagged full-length and truncated LAZY1 proteins, along with *in vitro* assays, indicated monocot and dicot LAZY1 proteins, as well as Arabidopsis AtLAZY2, 3 and 4, associate with the plasma membrane and, in some cases, microtubules and nuclei^4,5,20,28,29^. The LAZY1 TMD was needed for membrane localization of transiently expressed rice and maize LAZY1-GFP in onion^20,27^. But an AtLAZY1 truncation lacking the TMD still localized to membranes in tobacco^28^. In addition, Arabidopsis protein fractionation assays suggested AtLAZY1 is a peripheral membrane protein, not a transmembrane one^28^. Lastly, the AtLAZY1 N-terminal Region I, upstream of the TMD was found to be important for both plasma membrane localization and branch angle control, as illustrated by site directed mutagenesis^29^. AtLAZY1 Regions II and V are also essential for LAZY1-directed branch angle control^29^. Interestingly, in Arabidopsis, nuclear localization is not needed for LAZY1-mediated branch angle control^4^. The *lazy1* shoot phenotype was rescued by overexpressing a *LAZY1* sequence containing a mutation in the NLS, which was shown to prevent nuclear localization in tobacco^4^.

The last ~14 amino acids of the LAZY protein C-terminus (which is also called domain/region V, the Conserved C-terminus in LAZY1, or the CCL, as well as peptide VI) contains an Ethylene-responsive Amphiphilic Repression (EAR) transcriptional repressor motif and this region seems to be important for LAZY protein function in many species^3,5,8,29^. It was required to rescue Arabidopsis *lazy1;lazy2;lazy4* mutant root gravitropism phenotypes^8^. Overexpression of this sequence in the triple mutant resulted in upward growing roots^8^. Further, site-directed mutagenesis of several amino acids within the EAR motif in region V reduced or eliminated rescue of the *lazy1* and *lazy1;lazy2;lazy4* phenotypes^29,30^. Lastly, the EAR motif mediated interactions between wheat LAZY4/DRO1 protein and auxin signaling repressor TOPLESS, suggesting it is functional in this species^11^.

*LAZY1* transcription in rice is directly and positively regulated by the HEAT STRESS TRANSCRIPTION FACTOR 2D (HSF2D) protein upstream of auxin transport in response to gravistimulation^31^. Yeast-one-hybrid assays suggested *LAZY4*/*DRO1* expression in wheat is directly regulated by AUXIN RESPONSE FACTOR 1 (ARF1) transcription factors^11,31^. In addition, yeast-two-hybrid and BiFC experiments determined that the maize LAZY1 (ZmLAZY1) protein bound to both a protein kinase (ZmPKC) which may be involved in PIN localization at the plasma membrane, and to a nuclear-localized Aux/IAA auxin signaling repressor protein (ZmIAA17)^5^. However, species-wide interpretations of results from maize should be cautioned because in addition to its role in gravitropism and shoot architecture, *LAZY1* is also essential for tassel and ear development in maize^5^.

In contrast to the mechanistic knowledge about *LAZY1*, much less is known about *TAC1*. Its function in promoting wide lateral shoot angles is dosage dependent and light regulated^3,13–19,32–34^. *TAC1* expression has been detected in shoot tissues in peach, poplar, and Arabidopsis, with higher expression detected in apical regions^3,16,34^. Further, expression in Arabidopsis was induced by light and eliminated after prolonged darkness or application of photosynthetic inhibitors^18^. Additionally, the narrow branch angle phenotype in Arabidopsis *tac1* plants phenocopies the branch angles of wild type plants exposed to continuous shade or the chlorophyll biosynthesis inhibitor Norflurazon^18^. Lastly, *AtTAC1* overexpression could not fully rescue the Arabidopsis mutant phenotype and it only partially prevented upright reorientation of branches in wild type plants in response to darkness^3,18^. These findings suggest additional pathways promote outward shoot orientations in Arabidopsis and/or the existence of post-transcriptional regulation of *TAC1*.

Here we examined the functional relationship between *TAC1* and *LAZY1* in Arabidopsis at the molecular and genetic level as well as in connection with gravitropic responses. Our experimental results suggest that, while these genes have several commonalities, they also have distinct functions. In addition, while they do not appear to directly regulate each other’s expression, an indirect negative regulation of LAZY1 function via TAC1 at the protein level cannot be ruled out.

## Results

### GUS reporter constructs revealed *TAC1* and *LAZY1* have similar expression patterns

**Figure 1:**
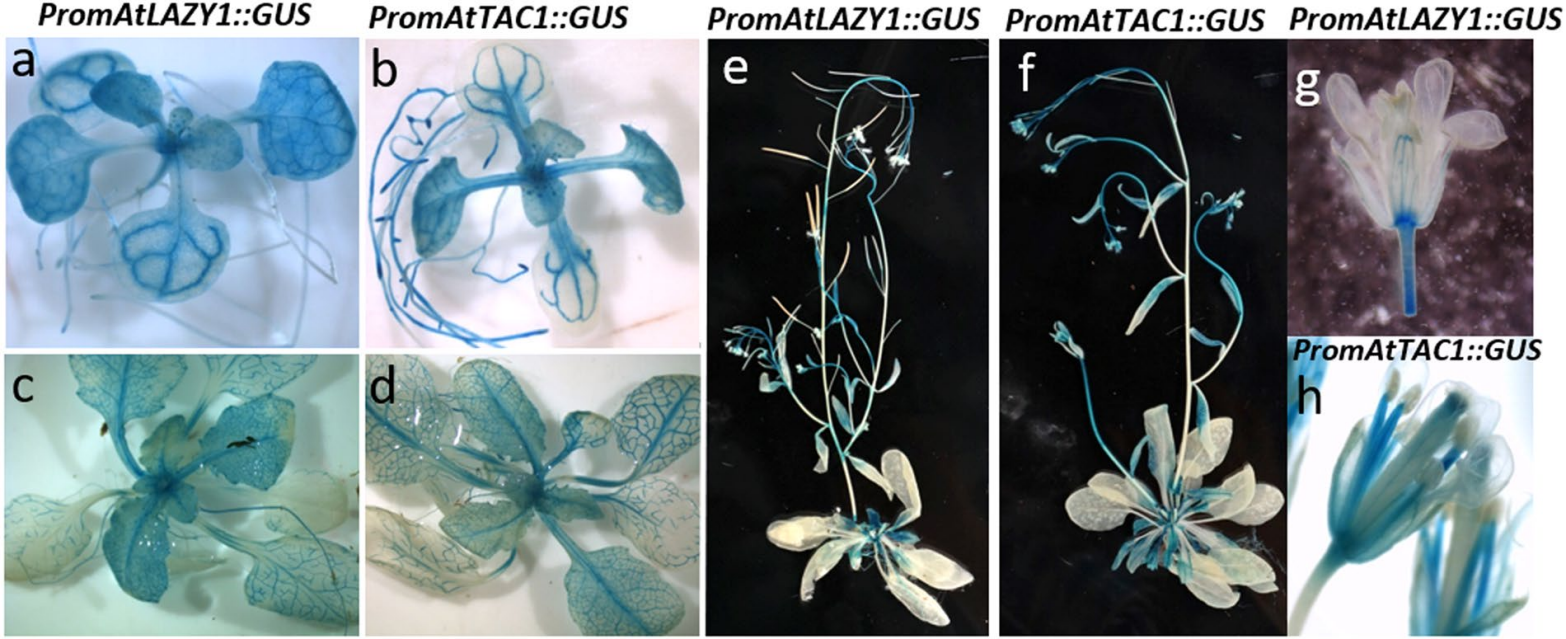
GUS staining of Arabidopsis containing *PromAtLAZY1::GUS* and *PromAtTAC1::GUS* reporter constructs revealed similar tissue-specific gene expression patterns. Two-week old *PromAtLAZY1::GUS* (**a**) and *PromAtTAC1::GUS* (**b**) seedlings. Four-week old *PromAtLAZY1::GUS* (**c**) and *PromAtTAC1::GUS* (**d**) seedlings. Mature At*PromLAZY1::GUS* (**e**) and *PromAtTAC1::GUS* (**f**) plants. Mature *PromAtLAZY1::GUS* (**g**) and *PromAtTAC1::GUS* (**h**) flowers.

Tissue-specific *TAC1* and *LAZY1* gene expression patterns were assessed in Arabidopsis plants throughout development using a *GUS* reporter system. Arabidopsis plants containing the Arabidopsis *LAZY1* promoter driving *GUS* (*promAtLAZY1::GUS*) previously revealed *LAZY1* promoter activity in shoots, vasculature, endodermis, petioles, and shoot apical meristem tissue, as well as a faint signal in the root which was generally restricted to root vasculature^4,7,8^. We generated an Arabidopsis line containing the *TAC1* promoter driving *GUS* (*promAtTAC1::GUS*) to compare spatiotemporal expression pattering between *LAZY1* and *TAC1*. We found *TAC1* promoter activity was highly similar to *LAZY1* in the shoot (Fig. 1; Supplementary Figures S1 and S2). Two- and four-week-old seedlings had strong expression in the vasculature of cotyledons and young leaves (Fig. 1a–d). Strong staining was consistently visible in the primary and most prominent (thicker) secondary roots of *promAtTAC1::GUS* seedlings as well as in lateral root tips (Fig. 1b; Supplementary Figure S2). In contrast *promAtLAZY1::GUS* seedling roots had fainter staining overall, although strong signal was sometimes observed in primary roots of older seedlings and adult plants (Fig. 1a; Supplementary Figure S1). Little to no expression was observed in mature *promAtTAC::GUS* and *promAtLAZY1::GUS* rosette leaves, but signal was present in rosette petioles as well as younger leaves to varying degrees (Fig. C-F; Supplementary Figures S1 and S2).

In mature plants, staining for *TAC1* and *LAZY1* expression was observed in shoots, with the highest signal in the apical regions (Fig. 1e,f, Supplementary Figures S1 and S2). As the distance from the shoot apices decreased, the signal became increasingly faint and was often absent from the base of the shoots (Fig. 1e,f, Supplementary Figures S1 and S2). For both *promAtTAC1::GUS* and *promAtLAZY1::GUS* plants*, GUS* expression in cauline leaves varied but was similar in intensity and localization and often strongest in the vasculature and at the base of the leaves (Fig. 1e,f, Supplementary Figures S1 and S2). *TAC1* and *LAZY1* reporter lines also had strong staining in newly emerging axillary shoots (Supplementary Figures S1 and S2).

The most noticeable difference in the localization patterns for the two genes was in the flowers, where *TAC1* was highly expressed in filaments but *LAZY1* signal was absent. GUS expression for both promoters was apparent in sepal vasculature, style, carpel base, and pedicels (Fig. 1g,h, Supplementary Figures S1 and S2). However, the *TAC1* signal was consistently stronger in these tissues (Fig. 1g,h; Supplementary Figures S1 and S2). On occasion, *promAtTAC1::GUS* signal was observed in the carpel valves and septum of developing siliques (Supplementary Figure S2).

### Arabidopsis *lazy1* branch phenotype was epistatic to the *tac1* branch phenotype

**Figure 2:**
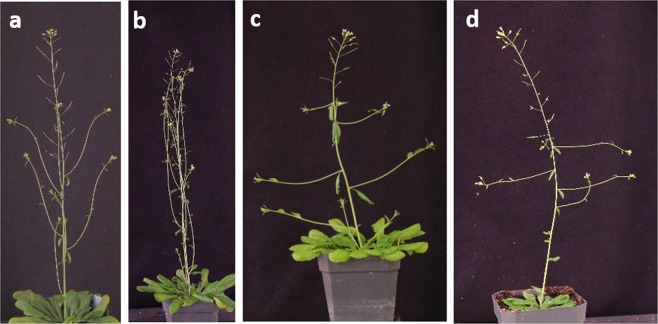
The *lazy1* branch phenotype is epistatic to *tac1*. (**a**) Col, (**b**) *tac1*, (**c**) *lazy1*, (**d**) *tac1; lazy1*.

The similarity between *TAC1* and *LAZY1 promoter::GUS* expression patterns along with the opposing phenotypes of *tac1* and *lazy1* Arabidopsis suggest a possible intersection between their control of branch orientation. To determine the combined effect of their non-functional alleles on shoot architecture, *tac1* and *lazy1* single mutants were crossed and the shoot orientation phenotypes of at least two hundred homozygous double mutants from multiple lines and generations were characterized. The *tac1;lazy1* double mutants clearly and consistently displayed a *lazy1*-like wide branch angle phenotype and could only be distinguished by genotyping (Fig. 2). Thus, the *tac1;lazy1* mutant phenotype suggests *lazy1* is epistatic to *tac1* with regards to branch orientation control.

### *tac1* mutants did not exhibit an altered gravitropic shoot bending response

**Figure 3:**
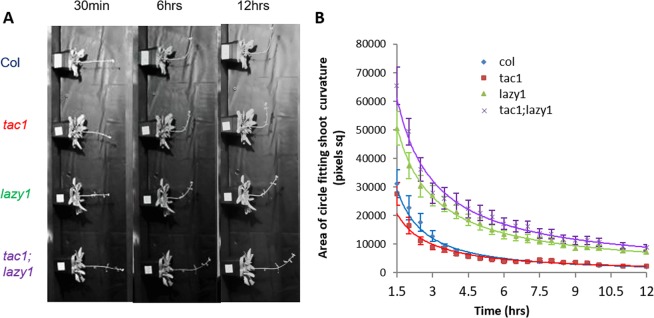
Gravitropic responses of wildtype (Col), *tac1*, *lazy1*, and *tac1;lazy1* Arabidopsis plants after a 90-degree reorientation. (**A**) Images of the same representative Col, *tac1*, *lazy1*, and *tac1;lazy1* plants at 30-minute, 6-hour, and 12-hour time points. (**B**) The rate of bending in response to reorientation represented by the decreasing area of circles that best fit the curve of each inflorescence meristem bend between 1.5 and 12 hours after reorientation. Each data point represents the average area from four independent experiments, each with five to nine plants per genotypes. Student’s t-test results found significant differences between the Col and *lazy1* and Col and *tac1;lazy1* values at every time point (p < 0.05). No significant differences were found between Col and *tac1* or *lazy1* and *tac1;lazy1* at any time point. Data points were fit with a power trendline and error bars indicate SEM.

Previous studies found that *lazy1* mutants had delayed gravitropic bending responses to shoot reorientation^4,5,21,26^. Here we tested if *TAC1* also has a role in gravitropic shoot bending. We hypothesized that *tac1* mutants might exhibit faster gravitropic bending responses than wild type and *lazy1* plants due to the more upward-oriented branch phenotype in *tac1* plants. We also wanted to test if the *lazy1* gravitropic response would be epistatic to *tac1*, just as the branch orientation phenotype was. To investigate these possibilities, we performed four separate 90° reorientation experiments with up to 30 Arabidopsis wild type Columbia-0 (Col), *tac1*, *lazy1*, and *tac1;lazy1* double mutant plants in pots (See Supplementary Figure S3 for our set-up). Plants were affixed to a custom-built rack and reoriented 90° under green light to eliminate phototropic interference and imaged in a single frame every minute for up to 12 hours to generate time-lapse videos, as exemplified in Video V1. To reduce the influence of plant size and shoot weight, all plants were a similar height and had only one primary shoot with short branches. Time-lapse videos were studied to assess if obvious reorientation differences existed between genotypes. We observed that the *tac1* plants fully reoriented, while *lazy1* and *tac1;lazy1* exhibited similarly slow bending responses compared to Col (Fig. 3, Supplementary Figure S3, Video V1). Obvious differences between *tac1* and Col plants were not detectable by eye. To quantitatively compare gravitropic bending rates, we initially measured the angle between plant shoot tips and the shoot trajectory. At 30- and 60-minutes post reorientation, *lazy1* and *tac1;lazy1* were very minimally less upright than Col (their average tip angles were closer to the 180-degree horizontal), and there was no significant difference between *tac1* and Col (Table S1). This agreed with our visual observation that *tac1* did not reorient faster and *lazy1* and *tac1;lazy1* responded equally slower than Col (Table S1). However, imaging a large field of view reduced the resolution of individual shoots and shoot tips, increasing variability and subjectivity when choosing anchor points for angle measurements. In addition, the simulated dark conditions resulted in extensive shoot tip circumnutation (clearly visible in Video V1). Consequently, tip angles from the images spanning the majority of the 12-hour time period did not always correlate with the degree of bending observed in the shoots. Therefore, we subsequently developed a novel approach to compare gravitropic responses over time in relation to shoot curvature, the most obvious response to phenotype. Using ImageJ software^35^ and images from twenty time points spanning 1.5 to 12 hours after reorientation, we extracted the area of circles that, when overlaid, best fit the curvature of each stem at the site of bending. This method enabled relative comparisons between genotypes for the later-stage bending response; as time from reorientation increases, shoots become more upright and the areas of the best-fitting circles decrease (Fig. 3). While this method works well to quantify the overall curvature of a stem, it is unable to assess possible differences during very early shoot lifting.

Using this ‘best-fit circle’ relative assessment method, we identified that *lazy1* and *tac1;lazy1* bending (curvature) responses were slower than Col and *tac1*. The circles fitting their shoots at all time points were consistently larger than those for Col and *tac1* (p < 0.05, determined by t-test) (Fig. 3). No differences were found between Col and *tac1* or *lazy1* and *tac1;lazy1* at any time point. The *lazy1* and *tac1*;*lazy1* genotypes exhibited a comparable delayed gravitropic curvature response, suggesting the loss of *TAC1* does not alter this *lazy1* gravitropism phenotype (Fig. 3). In addition, we found no differences in overall rate of reorientation between *tac1* and Col using this method (Fig. 3).

### Ectopic *LAZY1* expression causes an EAR-domain dependent curled leaf phenotype

**Figure 4:**
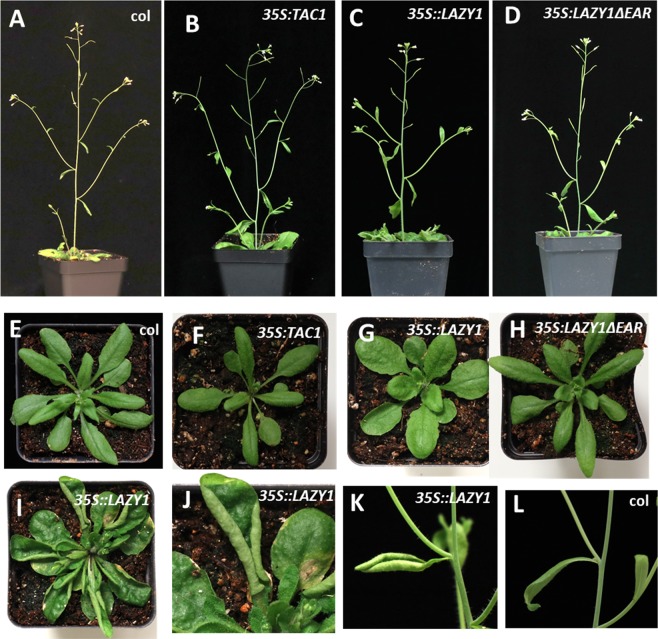
Overexpression of *LAZY1* resulted in leaf curl but not altered branch angles. Arabidopsis Columbia (**A,E**), *35 S::TAC1* (**B,F**), *35 S::LAZY1* (**C,G**), and *35 S:LAZY1∆EAR* (**D,H**) plants and young rosettes prior to bolting. (**I**) Mature *35 S::LAZY1* rosette with bolt removed (**J**) Enlarged image of curled rosette leaf from (**I**). (**K**) *35 S::LAZY1* node with a rare upward curled cauline leaf. (**L**) Columbia node with cauline leaf.

We previously reported that overexpression of *TAC1* in plum trees resulted in wider branch angles^17^. However, we also reported *TAC1* overexpression only partially complemented the *tac1* Arabidopsis mutant phenotype, and in 24-hour light or dark conditions *TAC1* overexpression in Col had little effect on branch angle^3,18^. In contrast, *35 S::AtLAZY4/DRO1* in Arabidopsis exhibited more vertically oriented lateral shoots as a result of narrower branch angles, as well as upward curled leaves^6^. Further, the overexpression of a *LAZY4/DRO1* sequence lacking the EAR motif (*35 S::AtLAZY4/DRO1*Δ*EAR*) did not result in these phenotypes^6^. Here, we investigated the effect of overexpressing the full-length *AtLAZY1* CDS (*35 S::LAZY1*) as well as a truncated sequence lacking the EAR motif (*35 S::LAZY1*Δ*EAR*) in the Col background (Fig. 4, Supplementary Figure S4). For comparison, *35 S::TAC1* in Col were grown alongside the *LAZY1* and *LAZY1*Δ*EAR* overexpression plants (Fig. 4B,F; Supplementary Figure S2). We found the *35 S::TAC1* plants exhibited a very slight, but significant increase in branch angle (Supplementary Figure S4). In addition, in contrast to the published *35 S::AtLAZY4/DRO1* phenotype, overexpression of full length *LAZY1* surprisingly did not produce obvious branch angle differences compared to Col (Fig. 4A–D). However, when measured, the *35 S::LAZY1* plants also had a slight increase in branch angle compared to Col (Supplementary Figure S4). Compared to Col, *35 S::LAZY1* plants also exhibited upward curling of rosette leaves, with some rolling up completely as they aged (Fig. 4I,J). Upward curled cauline leaves were also observed sporadically in the *35 S::LAZY1* plants but not in Col. These leaf phenotypes were comparable to those in *35 S::AtLAZY4/DRO1* plants^6^. Curled leaves were not observed in *35 S::AtLAZY1*Δ*EAR* or *35 S::TAC1* (Fig. 4). In addition, young rosette leaves from *35 S::LAZY1* plants were more rounded than the Col, *35 S::TAC1*, and *35 S::AtLAZY1*Δ*EAR* plants (Fig. 4E–H). The lack of a *35 S::LAZY1* branch angle phenotype, in contrast to the narrower branch angles exhibited in published *35 S::LAZY4/DRO1* lines, suggests possible spatial regulatory divergence regarding lateral shoot orientations, as *LAZY4/DRO1* primarily regulates root angle. However, the shared leaf curl phenotype, and absence of this phenotype in both *LAZY1ΔEAR* and *LAZY4/DRO1*Δ*EAR* overexpression plants, suggests some functional conservation, as well as highlights the importance of the EAR motif for *LAZY* family members.

### *lazy1* plants exhibit substantially elevated SA levels

**Figure 5:**
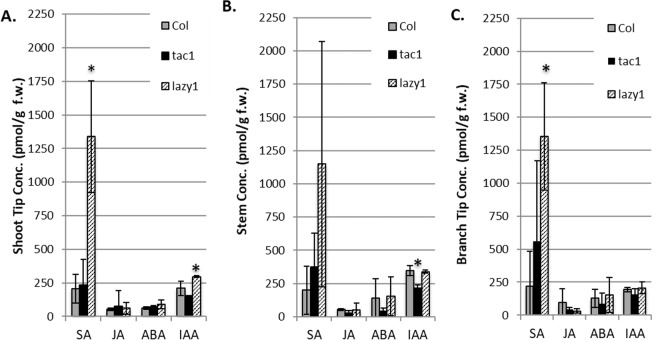
LC/MS/MS hormone analysis from shoot tissues. Hormones were extracted from (**A**) the shoot tip, (**B**) the stem immediately below the shoot tip segment, and (**C**) shoot apex of lateral branches. Bars represent standard deviation of biological replicates, each of which contained pooled tissue from multiple plants. *Indicates hormone concentration was significantly different than the same tissues from other genotypes according to ANOVA, p < 0.01 with Tukey’s ad hoc test.

Hormone concentrations and gradients play a key role in directing shoot architectures, including lateral shoot orientations. Prior studies established that *LAZY1* acts upstream of auxin transport^5,7,20,21,26,31^. Pillar peach trees (*tac1* mutants) were found to have elevated auxin concentrations in their shoot tips relative to standard cultivars^36^. Further, altered Abscisic Acid (ABA) localization in branches was associated with a weeping mulberry tree architecture, and *tac1* is epistatic to a weeping peach architecture^32,37^. To investigate if *TAC1-* or *LAZY*-mediated shoot orientations are associated with hormone concentrations, LC/MS/MS was performed on extracts from Arabidopsis *tac1*, *lazy1*, and Col shoot tips, stems (just below the tip), and branch tips from plants (Fig. 5; Supplementary Figure S5). Salicylic Acid (SA), Jasmonic Acid (JA), ABA, and auxin (IAA) concentrations were quantified. These tissues were chosen because they are regions where *TAC1* and *LAZY1* gene expression is highest.

In all three tissue types, SA levels were substantially higher in *lazy1* compared to both Col and *tac1*, with statistically significant increases in the primary shoot and branch tips (Fig. 5). Arabidopsis *tac1* plants had less free IAA than Col in both shoot tips and stems, with the latter being statistically significant (Fig. 5A,B). In addition, *lazy1* shoot tips had significantly greater IAA concentrations (Fig. 5A,B). No statistically significant differences in JA or ABA concentrations were detected between genotypes for the tissues we tested, although ABA levels in *tac1* were slightly lower in stems and branch tips (Fig. 5).

### Transcriptomes suggest *tac1* and *lazy1* mutants have altered gene regulation in response to gravistimulation

To compare influences of *TAC1* and *LAZY1* on transcriptional regulation, RNAseq transcriptome profiles were generated for inflorescence shoot tips from Col, *tac1*, and *lazy1* under normal conditions (t0) and after 45 minutes of gravistimulation by 90° reorientation (t45) (Supplementary Data File D1). Shoot tips were again used because *promoter::GUS* and qPCR studies revealed *TAC1* and *LAZY1* are highly expressed in shoot tips and often absent from the middle and base of primary shoots and branches (Fig. 1; Supplementary Figures S1 and S2)^3^.

**Figure 6:**
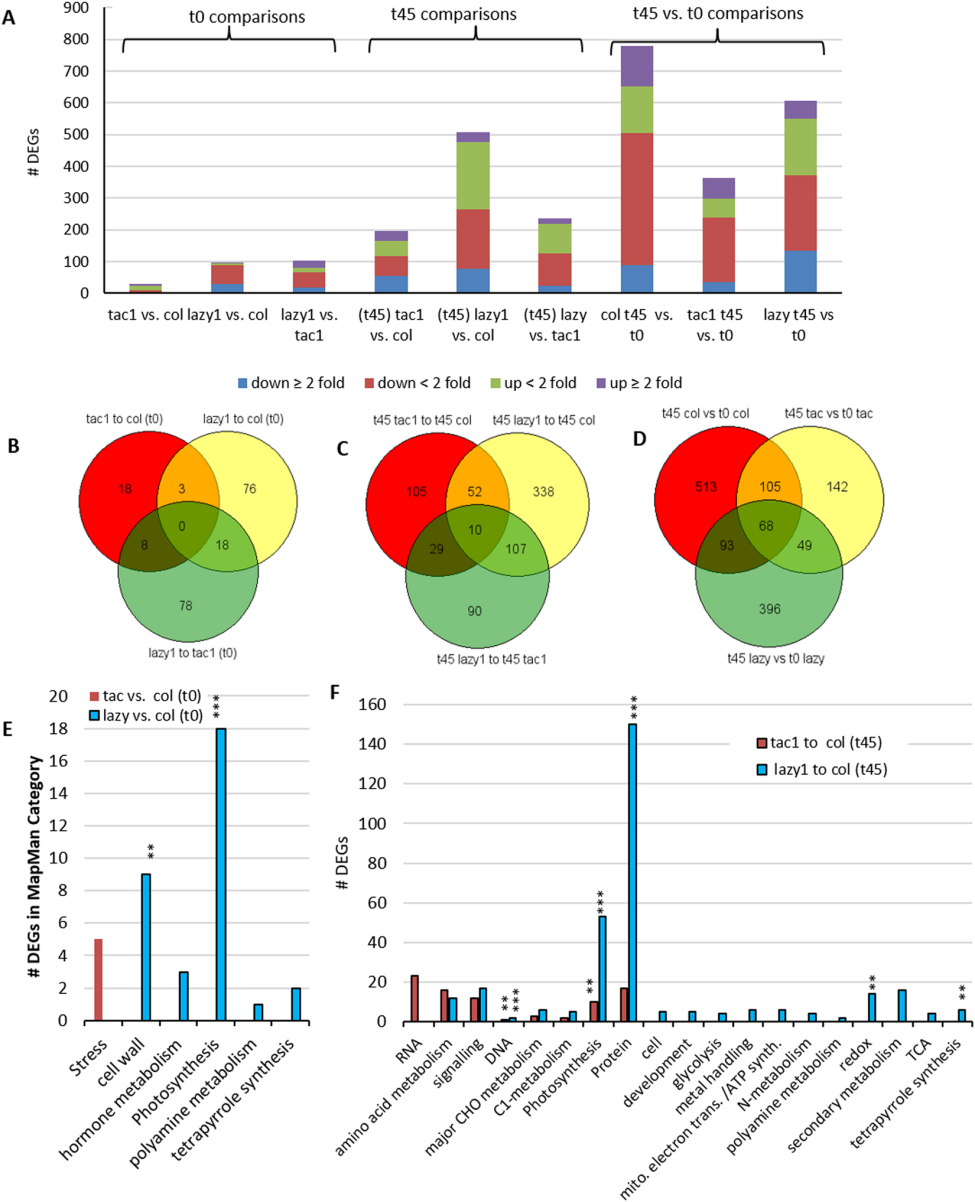
Differentially expressed genes (DEGs) between upright and gravistimulated Columbia (Col), *tac1*, and *lazy1* arabidopsis. (**A**) Numbers of DEGs from different comparisons of RNAseq data from upright (t0) and gravistimulated (t45) plants. (**B,C,D**) Venn diagrams indicating numbers of overlapping DEGs between t0, t45, and t45 vs. t0 comparisons. (**E,F**) Numbers of DEGs in significantly enriched MapMan gene categories for upright (**E**) and gravistimulated (**F**) comparisons. All categories have statistically significant representation of DEGs with a p < 0.05. ** indicates very high significance (p < 0.0001) and *** indicates extremely high significance (p < 10e-10).

After comparing expression profiles of each mutant to Col under the same conditions (normal or gravistimulated), a combined total of 829 differentially expressed genes (DEGs) were identified (Supplementary Data File D2). Fewer than 200 of these genes had expression changes greater than 2-fold, and very few DEGs had changes greater than 5-fold (Fig. 6A; Supplementary Data File D2). Most of the largest differences of expression were in response to gravistimulation. Interestingly, although no gravitropic bending differences between *tac1* and Col were detected, in response to gravistimulation Col shoot tips had over two times the number of DEGS than *tac1* (Fig. 6A, Supplementary Data File D2).

Due to their opposing phenotypes and sequence similarity, we hypothesized that TAC and LAZY1 may act through common pathways. Accordingly, we anticipated that *tac1* and *lazy1* transcriptomes might have overlapping of DEGs, and potentially some with opposite directional changes. However, there were only three DEGs in common between the genotypes with upright orientations (Fig. 6B–D, Supplementary Data Files D2 and D3). They were *AtLFNR2*, *CSP41S*, and *HTB4* and they all had minimal fold changes, the changes were not in opposite directions, and the three genes have no known functional relationships to each other (Supplementary Data Files D2 and D3). Similar results were observed for the DEG comparisons from the gravistimulated plants (Fig. 6C,D; Supplementary Data Files D2 and D3).

A very small number of DEGs stood out as exhibiting exceptionally large differences in expression between genotypes. These included the uncharacterized gene *At3g01345*, upregulated nearly 400-fold in upright (t0) *tac1* and *METHIONINE RESPONDING DOWN 1* (*MRD1*), repressed over 300-fold in upright (t0) *lazy1* (Supplementary Data File D2). Secondary cell wall formation genes *TRACHEARY ELEMENT DIFFERENTION* 6 (*TED6*) and *TED7* were also greatly downregulated in the gravistimulated (t45) *lazy1* tissues (−82 and −43-fold, respectively) but not in t45 Col (Supplementary Data File D2). Another notable result was *LAZY1* was not differentially expressed in *tac1* nor was *TAC1* differentially expressed in *lazy1* under either normal or gravistimulated conditions (Supplementary Data File D2). Further, *TAC1* and *LAZY1* were not differentially expressed in Col shoot tips in response to gravistimulation (t45 Col vs. t0 Col) (Supplementary Data File D2). This is consistent with a prior report of *LAZY1* expression increasing only after several hours of 90° reorientation in rice seedlings^31^. Thus, our collection time may have been too early after 90° reorientation to detect differential expression of *TAC1* and/or *LAZY1*. In addition, the transcriptome analyses for gravistimulated plants come with the caveat that they are from whole shoot tips; any asymmetric (upper vs. lower) expression differences in the shoot tips would not be detectable.

Despite the higher concentration of SA in *lazy* shoot tips and stems, *DEGs* associated with SA were not identified in *lazy1* plants grown under normal conditions (Supplementary Data File D2). However, there was a 3-fold decrease in *BSMT1* expression in gravistimulated *lazy1*. This methyltransferase is induced by biotic and abiotic stress to produce methyl-salicylate (MeSA), a mobile signal for systemic acquired resistance^38^. *BSMT1* downregulation might be a response to a negative feedback mechanism triggered by high SA levels. Yet, this same gene was upregulated 8-fold in in gravistimulated Col (compared to upright Col) (Supplementary Data File D2). SA-degradation gene *MO1* was also upregulated ~8-fold in t45 Col, as well as *WAK1*, a cell wall associated kinase known to respond to SA. Another DEG with a SA connection (*ADH2*) was downregulated in the t45 *lazy1* transcriptome, but to a lesser degree. *ADH2* expression is reduced in response to high SA and, interestingly, *adh2* mutants exhibit dwarfing and dark-grown seedlings do not elongate their hypocotyls (phenotypes reported by ABRC; arabidopsis.org)^39^. In the t45 Col samples, a few additional SA-responsive genes were also slightly differentially expressed (Supplementary Data File D2).

A MapMan categorical analysis of the DEGs from each comparison revealed both expected and unexpected enrichments. This included an enrichment of only stress-related genes for upright *tac1* plants compared to Col (Fig. 6E, Supplementary Data File D4). A similar enrichment was reported for *tac1* (pillar) peaches^17^. Supporting the known connection between *LAZY1* and auxin transport, and in accordance with the *lazy1* mutant phenotype, t0 *lazy1* transcriptomes (when compared to t0 Col) were enriched for DEGs associated with hormone metabolism, the cell wall, and photosynthesis, among other categories (Fig. 6E, Supplementary Data File D4). This included downregulated auxin efflux transporters *PIN3* and *PIN4*, auxin responsive *SAUR16*, multiple expansins (*EXP3*, 5, and 8), *FASCICILIN-LIKE ARABINOGALACTANS* (*FLA2* AND 11), wall degradation enzymes (*GH98B* and a pectin lyase), a cellulose-synthase-like gene (*CSLC4*) and 18 photosynthesis-related genes (Supplementary Data Files D2 and D4).

MapMan categorizations of DEGs from gravistimulated mutant to Col comparisons and t45 vs. t0 plants of the same genotype identified enrichment in additional gene categories, (Supplementary Figures S4, S6 and Supplementary Data File D4). These categories included various types of metabolism as well as the general ‘Protein’ and ‘RNA’ categories (Fig. 6F, Supplementary Figures S4, S6 and Supplementary Data File D4).

## Discussion

IGT family genes *TAC1* and *LAZY1* have opposite but essential roles in directing lateral shoot orientations in vascular plants. *TAC1*, which is upregulated in response to light, promotes outward shoot orientations (via establishing wide branch or tiller angles). *LAZY1*, acting in the gravitropism pathway upstream of lateral auxin transport, promotes upward shoot orientations (via establishing narrow branch or tiller angles). We previously proposed that *TAC1* and *LAZY1* may act in the same pathway but one (or possibly both) can regulate the other’s function^2^. This was logically based on their opposing mutant phenotypes, the presence of *LAZY* genes in primitive plants and the apparent evolutionary loss of the EAR motif in *TAC1*, and gene dosage-dependent phenotypes in *tac1* Arabidopsis and peach heterozygotes^3^. However, genetic interactions between *TAC1* and *LAZY1* as well as a direct connection between *TAC1* and gravity response have not been established. Here, we found that *LAZY1* and *TAC1* were both expressed in similar shoot tissues at the same stages of development, as assessed using GUS reporters. Second, we found that *lazy1* was epistatic to *tac1* as double *tac1;lazy1* mutants displayed a *lazy1* branch phenotype. In addition, loss of *TAC1* had no detectable impact on gravitropic bending or curvature responses, and the double *tac1;lazy1* mutant showed very similar bending dynamics to *lazy1* alone. Lastly, auxin levels were also inverse in the two mutants with *lazy1* shoot tips exhibiting slightly higher IAA levels and *tac1* shoot tips having lower IAA levels. While these results are consistent with TAC1 potentially serving as a negative regulator of LAZY1, the collective data were not entirely consistent with a simple negative regulatory model.

If TAC1 was a direct negative regulator of LAZY1, we might identify opposite directional changes of common DEGs in the respective mutants, which we did not observe. In addition, *LAZY1* expression was not differentially expressed in *tac1* plants nor was *TAC1* expression altered in *lazy1* mutants. Further, although *TAC1* and *LAZY1* overexpression in some species significantly enhanced branch/tiller angles, this was not the case in Arabidopsis^13,16,17^. *35 S::TAC1* lines were only minimally wider than Col while *35 S::LAZY1* also exhibited slightly wider branch angles, a strong contrast to previously reported narrow branch angle phenotype of 35 S::*LAZY4/DRO1* Arabidopsis^6^. Only the curled leaf phenotype of *LAZY4/DRO1* overexpression was recapitulated when using *LAZY1*. This result may originate from minor functional differences between species for both genes and among *LAZY*-family members. While the branch angle directional change in *35 S::LAZY1* contradicts known function, a recent publication demonstrated inherent branch angle inconsistencies when expressing a *LAZY1* transgene in Arabidopsis^29^. Together these findings suggest TAC1 and LAZY proteins and their interactions may be subject to complex regulatory mechanisms.

Although the precise nature of the relationship between these two genes remains unclear, the data presented here provide several important insights. First, relatively few genes were differentially expressed in *tac1* and *lazy1*. Second, the majority of the DEGS were downregulated. Thus, if the LAZY1 protein can act as a repressor via its EAR motif, it likely targets few genes, and may repress one or multiple repressors. Also, potential transcriptional repression by LAZY1 in Arabidopsis is likely secondary to its gravitropism role, as nuclear localization was not required for maintaining branch orientation in this species^4^. Third, our RNAseq data are consistent with a role of LAZY1 in influencing gravitropic responses. Genes repressed in *lazy1* plants included cell wall-related genes such as expansins and FLAs (whose expression was downregulated in *lazy1*), thereby promoting asymmetric cell elongation in shoots for positioning purposes. The lateral auxin transport genes *PIN3*, *PIN4*, and auxin influx gene *AUX1* were also downregulated in *lazy1*.

Interestingly, one gene showed extreme differential expression. *MTO 1 RESPONDING DOWN1* (*MRD1*) was repressed by over 300-fold in upright *lazy1* shoot tips. *MRD1*, is minimally characterized as of now, with the exception that it is downregulated in *mto1-1* mutants that over-accumulate methionine. However, this gene in the root system architecture mutant *agb1-2* was found to be co-expressed with *At3g01345*, the most highly induced gene in shoot tips from upright *tac1* plants^40^. Connections between *MRD1*, *At3g01345, TAC1*, and *LAZY1* warrant further investigation, as the functions of both are not understood.

One of our most surprising findings, and one we cannot at this time explain, was the tremendous increase in SA concentrations (~5 fold) in shoot tips, stems, and branch tips of *lazy1* mutants. SA is primarily associated with biotic stress and no prior connections between this hormone and any *lazy* mutants have been reported. However, several studies have shown that SA can regulate shoot elongation. SA accumulation in response to cold temperatures inhibits cell elongation and reduces plant growth, and SA signaling (but not concentration) is important for petiole elongation in response to shade^41,42^. It’s possible that, in addition to auxin transport impairments, the *lazy1* branch angle phenotype is connected to reduced cell elongation in abaxial branch tissues as a result of high SA concentrations. The Arabidopsis *LAZY1* promoter region also has several SA responsive W-Box and WLE1 elements — (T)TGAC(C/T) and TGACA respectively — suggesting SA may have a direct role in regulating *LAZY1* expression^43,44^. However, our RNAseq data uncovered DEGs more tangentially, rather than directly, related to SA biosynthesis and signaling. Given these results and the presence of SA-responsive elements, connections between *LAZY1* and SA warrant further exploration.

In summary, the data described here highlight molecular and genetic aspects of *TAC1-* and *LAZY1* function and their relationship to each other. Recent findings linking LAZY proteins to PIN3 localization via physical interactions with BRX-domain proteins suggest a possible mechanistic area where TAC1 and LAZY1 function could intersect^30,45^. One possibility is that *TAC1* acts to integrate light perception into an ancient *LAZY1-*mediated gravity response pathway, thereby optimizing shoot positions for light capture. However, extensive experimentation is needed to build a comprehensive understanding of their independent and/or interdependent roles in regulating lateral shoot orientations in plants. The data provided here fills in some key knowledge gaps and provides new directions for future studies on *TAC1* and *LAZY1* branch angle control.

## Materials and methods

### Cloning

The *promAtTAC1::GUS* construct was generated by cloning a 2-kb fragment of the *AtTAC1* promoter sequence, including the 5’ untranslated region (5′UTR), upstream of the *GUS* CDS in a pBI101 vector, using *SalI* and *SmaI* restriction sites. The *35 S::LAZY1* plasmid was made by amplifying the *AtLAZY1* CDS from Arabidopsis cDNA using the forward primer 5′-CGA GTC GAC ATG AAG TTT TGG GGC TGG-3′ and reverse primer 5′-AAT GGA TCC TTA CAG TTC CAA CAC GAA ATA GTC TTC-3′ and cloning it into pCR8/GW/TOPO. After sequence confirmation, the CDS was inserted downstream of the 35 S promoter in 35S-pBIN-AFRS (a modified pBINPLUS/ARS vector) using the *SalI* and *BamHI* restrictions sites^46^. Similarly, the *35 S::LAZY1∆EAR* construct was made by amplifying the *AtLAZY1* CDS, using a reverse primer (5′-AAT GGA TCC TTA ATA GTC TTC GTC TGT CTT GAT CCA G-3′) that excluded the sequence for the last 15 amino acids of the full-length protein.

### Plant material, Genotyping, and Branch Angle Measurements

The Arabidopsis *tac1*, *lazy1*, *promLAZY1::GUS, and 35 S::TAC1* plants were previously described^3,4,18^. The *tac1;lazy1* homozygous double mutant lines were generated by crossing the single mutants. Genotyping for *tac1* and *lazy1* was performed using standard PCR methods. To detect the *lazy1* mutant allele, the primer AtLAZY-Fwd (5′-CAA GGT TTT TAC AAC ACA GAG CAA) was with AtLAZY-T-DNA Rev (5′-ATA TTG ACC ATC ATA CTC ATT GC) primer. To detect the wild type *LAZY1*, AtLAZY-Fwd was used with At-LAZY-genotype-Rev (5′ GCT GAC ACC CAT CAT AAT GCT T). The mutant *tac1* allele was detected using AtTAC1-F-genotype (5′TCA ATT GTT CGT GTG CGT TT) and AtTAC1-R-genotype (5′AAA AGC TCC GCA AGT GTT GT). Wild type *TAC1* was detected with AtTAC1-F-genotype and AtTAC1mut-Rev (5′TTA AAA ACG TCC GCA ATG TG). The *35 S::LAZY1*, *35 S::LAZY1∆EAR*, and the *promAtTAC1::GUS* plants were generated by transforming their respective constructs, described above, into Arabidopsis (cv. Columbia) via agrobacteria-mediated floral dip transformation and kanamycin selection. Multiple transgenic lines were phenotyped and/or stained and average lines were used for characterizations. All plants were grown in either 2- or 4-inch pots with one plant per pot and 16-hour light (~100 µmol m^−2^ s^−1^) in growth chambers set to ~22 °C. Branch angle measurements for Col, *35 S::TAC1* and *35 S::LAZY1* plants were determined by measuring from the tip of each branch (just below the floral cluster) to the branch node and then up the stem to the node above each branch.

### GUS staining

Tissues for GUS staining were harvested in cold 90% acetone prior to a 20-minute room temperature incubation. Afterwards, tissues were rinsed with a staining buffer solution containing 50 mM NaHPO_4_ buffer (pH 7.2), 0.2% triton, 0.5 mM potassium ferrocyanide, and 0.5 mM potassium ferricyanide. Next, samples were submerged in staining buffer containing a 2 mM final concentration of X-gluc, vacuumed on ice for ~20 minutes, and incubated in dark for up to 16 hours at 37 °C. The next day, tissues underwent 30-minute incubations in the following solutions: 20%, 30%, and 50% ethanol, FAA, and then 70% ethanol. Several warm 95% ethanol washes followed until tissues cleared and samples were stored in 95% ethanol until imaged using a digital camera or a Nikon SMZ-800N stereomicroscope with a Nikon DS-Fi3 camera.

### Gravitropism experiment

For the gravitropism experiments, on four different dates 4-5-week-old plants in 2″ square pots with a single inflorescence shoot approximately ~10–12 cm tall were placed in a custom-built rack at the same time of day, adapted to dark conditions under green light in a sealed room for one hour, and then rotated clockwise 90^°^ to stimulate shoot bending. Prior to dark treatment, a metal pin was placed on the right side of each shoot, between the tip and base, to minimize initial shoot dropping. Between six and nine plants of each genotype were included for each experiment and the placement of each genotype was randomized. A total of 28 Columbia, 27 *tac1*, 30 *lazy1*, and 23 *tac1;lazy1* plants were used for quantification. Images were taken every minute for between 12 and 36 hours after rotation with a Canon EOS REBEL T3 digital camera using a time-lapse intervalometer shutter release control. A timer, counting from zero, was started immediately after reorientation and placed in the field of view of the camera to ensure accurate correlations between images and time after reorientation. Time-lapse videos were generated using Microsoft Movie maker or Microsoft Video Editor. To quantify bending, images from twenty time points between 1.5 hours and ending at 12 hours (at 30- or 60-minute intervals) were selected. For plants on these images, ImageJ was used to draw circles that best fit the curvature at the bending point in the stem and then determine the area of that circle. A total of 2,160 measurements were taken. For each time point, a Student’s t-test was used to test for statistically significant differences between the average area for each genotype to each other.

### RNA isolation for transgenic plants and qPCR analyses

Total RNA was extracted from Arabidopsis leaves using the Zymo Research Direct-zol RNA MiniPrep kit (Irvine, CA). qPCR was conducted using 50 ng total RNA per sample using the iTaq Universal SYBR Green One-Step Kit (BioRad, Hercules, CA) in a 15 µl total reaction volume in a 384 well plate on a BioRad CFX384 Touch with the following settings: 50 °C for 10 min for the RT step followed by a 95 °C 1 minute denaturation and then 35 cycles of 95 °C for 10 seconds and 60 °C for 20 seconds. A melt curve was subsequently performed to confirm the presence of only one amplicon. *LAZY1* primers were Fwd: TCC GGG AGA ATA GCA AAG AGC CAT and Rev: 5′ TGT ATT GCT TGG GTC CTG CGA A; *TAC1* primers were Fwd 5′AGC TGG TCA TGT CAA AGT CCA and Rev: 5′ TCA CAG TTC CGA GTT GGC TTG T). For *35 S::LAZY1*, *35 S::LAZY1∆EAR*, and *35 S::TAC1* lines between three and six biological reps were tested, each with three technical reps. Samples were analyzed for relative expression based on a standard curve using Columbia RNA.

### Hormone analysis

Phytohormone extraction and LC/MS/MS methods were adapted from an established protocol^47^. Approximately 1 cm samples were collected from the tip of the primary inflorescence shoot, 1 cm below the tip, and the tip of the first sub-apical branch of Arabidopsis Columbia, *lazy1*, and *tac1* plants (Supplementary Table S2). At the time of collection, the plants were approximately 12 cm tall and had only one shoot and no more than three branches. Three biological replicates were generated, each containing a pool of samples from 12-15 plants. These samples were flash-frozen in liquid nitrogen and the fresh weight of each pool was recorded. Phytohormones were extracted by grinding samples with a drill-driven blue pestle in buffer composed of 80% MeOH, 20% H_2_O, 0.1% formic acid, 0.1 g/L butylated hydroxytoluene, and internal standards of 100 nM [D_6_]-ABA (Toronto Research Chemicals) and 100 nM [D_7_]IAA (CDN Isotopes), using 1 mL of extraction buffer per 100 mg of sample. Samples were then rotated at 4 °C for 18 hours, centrifuged to pellet debris, the supernatant was filtered through a 0.2 m centrifugal filter unit (Millipore), and transferred to an autosampler vial. Samples (10 µL injection) were separated on an Ascentis Express C18 column (5 cm ×2.1 mm, 2.7 µm pore size (Supelco) using a linear gradient of 0.1% Formic Acid (solvent A) to 100% methanol (solvent B) with a flow rate of 0.4 mL/min, column temperature of 50 °C, and a 5 minute program. The separated samples directly interfaced with a Quattro Premier XE (Micromass Technologies) tandem quadrupole mass spectrometer via electrospray ionization. Capillary voltage, cone voltage and extractor voltage were 3 kV, 25 V, and 5 V, respectively. Source temperature was 120 °C and desolvation temperature was 350 °C. Cone gas flow was 50 L/hr, desolvation gas flow was 600 L/hr, and collision gas flow was 0.15 mL/min. Optimized detection parameters for each analyte is listed in Supplementary Table S2. Detection was performed by multiple reaction monitoring (MRM) in positive ion mode for IAA and IAA conjugates and negative ion mode for ABA, JA, and SA. Data acquisition and calculation was performed in Masslynx 4.1 software. A 4-fold dilution series from 1 µM to 1 nM was used to generate a standard curve, which then allowed for quantification of each phytohormone by peak area rations related to their corresponding internal standard ([D_6_]-ABA or [D_7_]-IAA).

### Transcriptome tissue collection

Arabidopsis plants containing only a single inflorescence shoot, with a height of ~15-18 cm, and no more than 3 lateral shoots (each no larger than ~1 cm) were used for expression profiling. Prior to collection, plants were left for at least 12 hours in a dark chamber with wooden stakes to support primary shoots to ensure they would be vertical at the time of collection. Primary shoot tips from upright (t0) plants and plants after 45 minutes of gravistimulation in the dark by a 90° reorientation (t45 plants) were collected on the same day. The t0 collections were at ~10 AM and t45 collections were at ~10:45 AM (having been reoriented at ~10 AM). From all plants, the upper most ~1 cm of primary shoot apical tissue (minus any leaves, flowers, or opening buds) was harvested and immediately frozen in liquid nitrogen. When t45 plants were reoriented to 90 degrees, their shoots were supported by microfuge tube racks to keep them in the same position, relative to the rosette, as when upright.

### RNA extraction for transcriptomes, RNA sequencing and transcriptome analysis

Total RNA was extracted from apical tissues using the Qiagen Plant Mini RNeasy kit (Germantown, MD) including a DNAse treatment with Invitrogen Turbo DNA-free (Carlsbad, CA). Approximately 2 μg of total RNA for each sample was sent to the Cornell Weill Medical Genomics Resources Core Facility (New York, NY, USA) where RNA TruSeq. 50 bp unpaired barcoded libraries were prepared for each and sequenced with nine libraries per lane on an Illumina HiSeq. 2000. Raw sequencing data, now available in the NCBI GEO database under accession GSE147254, was uploaded to CLC Genomics Workbench Version 9.5.3 (Qiagen, Redwood City, CA) and trimmed based on a QC limit of 0.05, an ambiguity max of 2 nucleotides, and a minimum length of 40 nucleotides. The number of reads for each tissue sample before and after trimming can be found in Supplementary Table S3. Using the CLC RNAseq analysis tool trimmed reads were aligned to the arabidopsis genome (TAIR10) with the following parameters: 2 maximum mismatches, a minimum length fraction of 0.9, a minimum similarity fraction of 0.8, a nonspecific match limit of 10, a minimum exon coverage fraction of 0.2, and 10 minimum reads. Expression values were reported as RPKM. Next, the RNAseq Experiment function was used to perform differential expression analysis. Five separate experimental comparisons were performed. A multiple comparison of all three types of t0 plants (Col, *tac1*, and *lazy1*) with three biological replicates for each. A multiple comparison of the t45 samples for all three plants, which included 4 biological replicates of Col and *lazy1* and 5 biological replicates for *tac1*. Individual analyses, comparing t0 to t45 samples of the same genome were also performed. All five experimental analyses were normalized by quantiles and then a proportion-based statistical analysis with Baggerley’s test as performed. Bonferroni corrected p-values and FDR-corrected p-values were also determined. Data sets for all comparisons containing DEGs with an FDR p-value correction ≤ 0.05 were then annotated using TAIR10 gene info and descriptions. For each of the five comparisons, the normalized relative fold-change values for genes with an FDR p-value ≤ 0.05 were used to identify significant MapMan Categories using the BAR Classification SuperViewer (https:bar.utoronto.ca)^48,49^.

Video V1. Time-lapse video of the 90° reorientation gravitropism experiment performed on May 5^th^, 2014. Video covers ~39 hours immediately following reorientation and is sped up 50×, with each image duration lasting 0.02 seconds. Shoot circumnutation is visible for some plants within the first 10 seconds (~2.5 hours into the experiment) and increases throughout the duration of the video. A key indicating the genotype of each plant can be found in Supplementary Figure S3.

## Supplementary information

Video V1.

Supplementary Figures and Tables.

Dataset 1.

Dataset 2.

Dataset 3.

Dataset 4.
