# Supplementary material for: Opposing influences of TAC1 and LAZY1 on Lateral Shoot Orientation in Arabidopsis
Source: Sci Rep. 2020 Apr 8;10:6051. doi: 10.1038/s41598-020-62962-4 (PMC7142156; doi:10.1038/s41598-020-62962-4)
Supplement: Supplementary file 2 — Supplementary Figures and Tables. [file 41598_2020_62962_MOESM2_ESM.pdf]

SUPPLEMENTARY DATA FOR

Title: Opposing influences of *TAC1* and *LAZY1* on Lateral Shoot Orientation in Arabidopsis

Courtney A Hollender[1,*], Joseph L Hill Jr.[1], Jessica Waite[2,3], and Chris Dardick[2]

[1]Department of Horticulture, Michigan State University, East Lansing, MI, 48824, USA.

[2]USDA-ARS Appalachian Fruit Research Station, Kearneysville, WV 25430, USA.

[3] Current Address: Washington State University Tree Fruit Research and Extension Center, Wenatchee, WA 98801

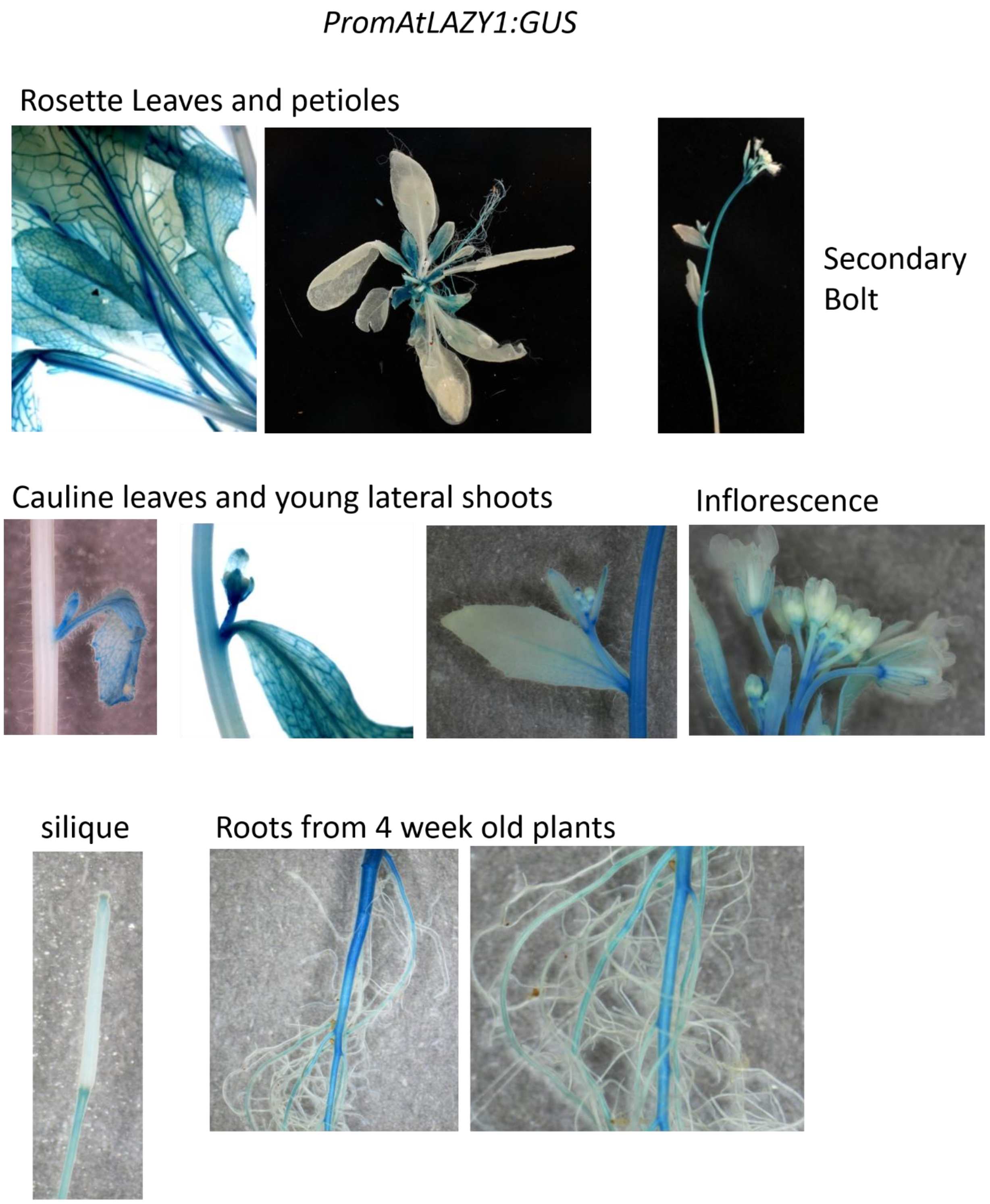

Supplementary Figure S1. GUS stained *PromAtLAZY1::GUS* plant tissues.

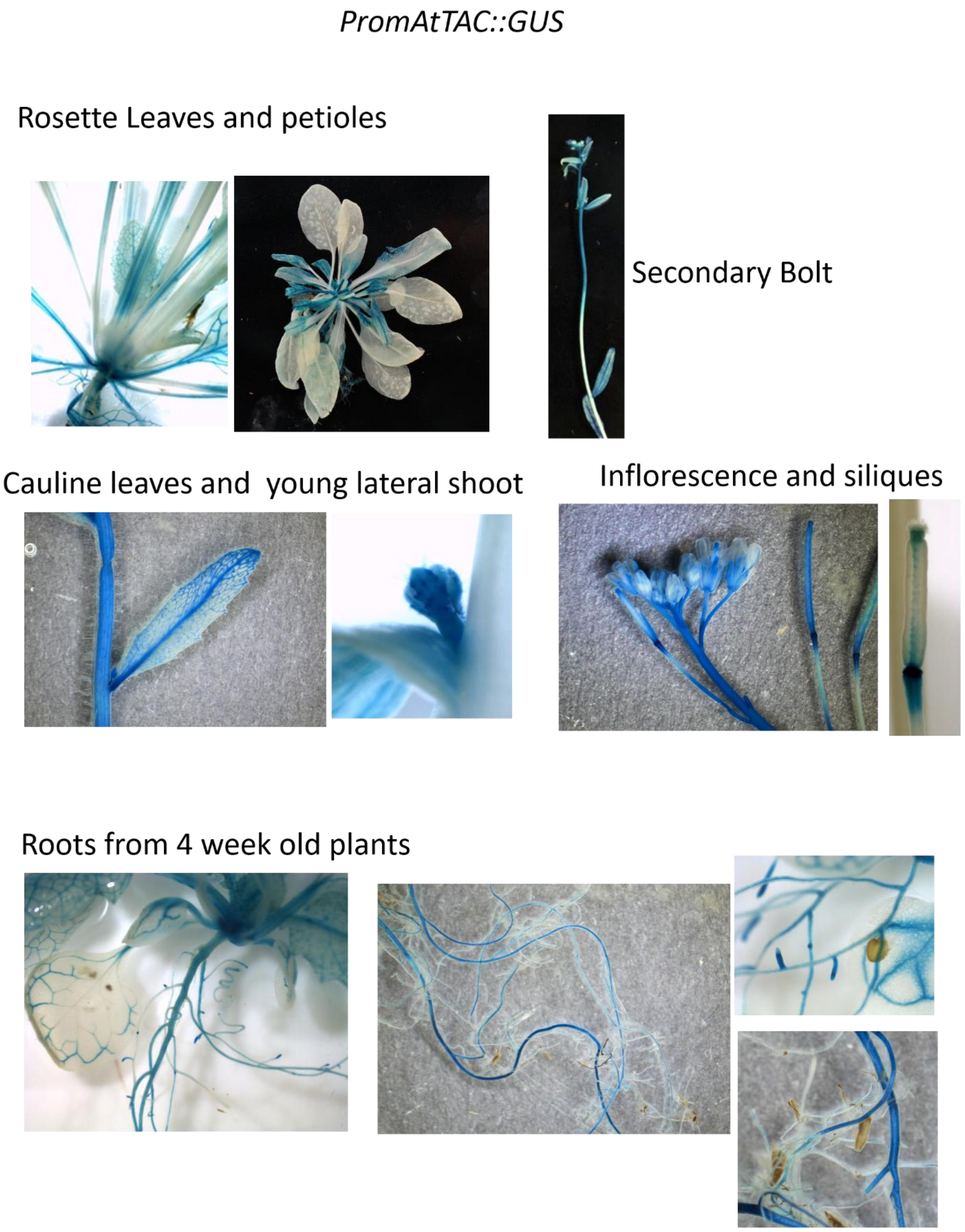

Supplementary Figure S2 GUS stained *PromAtTAC1::GUS* plant tissues.

A. Reorientation Experiment Set-up

1) Pots attached to custom-built rack

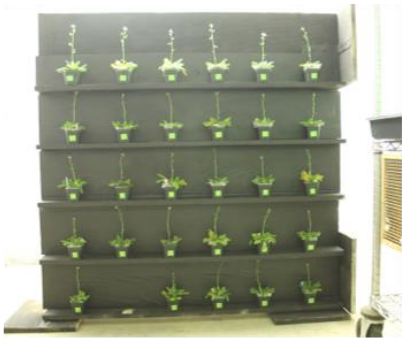

2) Acclimated to Dark (green light) for 1 hour

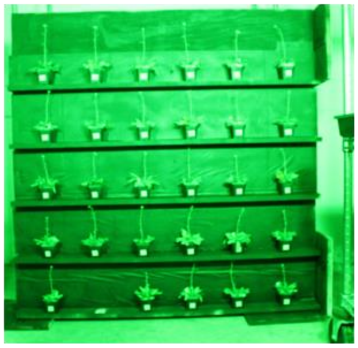

3) Rack rotated 90º and imaging started

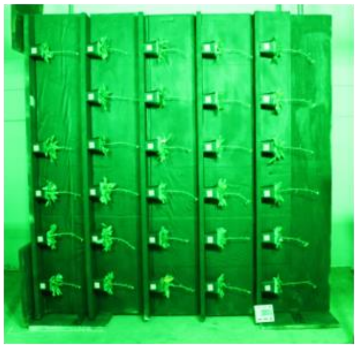

B. Initial and twelve-hour time point images for May 5th, 2014 experiment, converted to black and white for better visualization

1 minute after reorientation

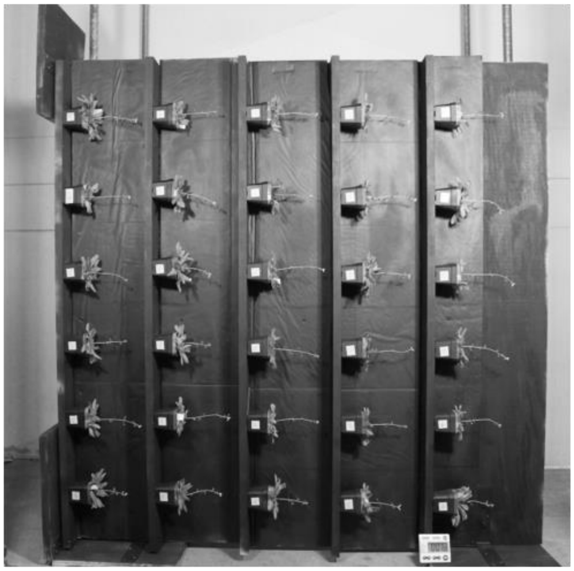

12 hours after reorientation

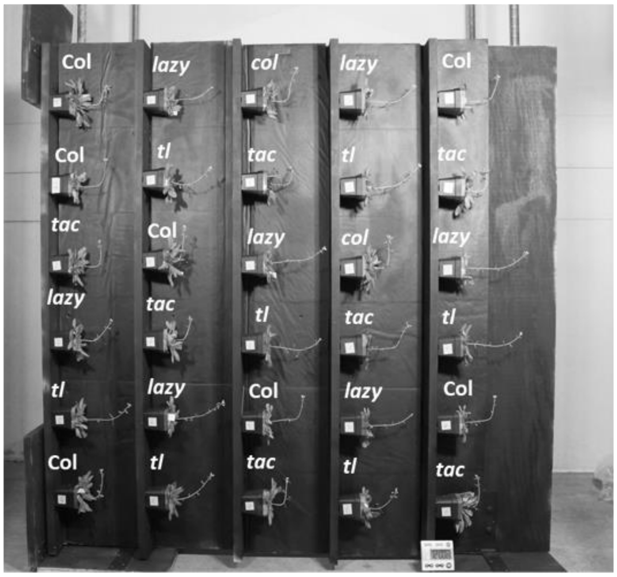

Supplementary Figure S3. Gravitropism reorientation experimental design. (A) Images from one of four reorientation experiments illustrating the experimental design. Plants in 2-inch pots were affixed to a custom-build rack and acclimated for 1 hour in green light (a proxy for dark conditions) before the rack was reoriented 90º clockwise. (B) Images of plants from a different reorientation experiment at 1 minute (left) and 12 hours (right) after reorientation. These images were converted to black and white for visual clarity. The genotype of each plant is written above each pot at the 12-hour time-point. LAZY1 represents *lazy1*, TAC represents *tac1*, TL represents *tac1;lazy1*, and Col represents wild type Columbia. A time-lapse video of this specific experiment can be found in Supplementary Video V1.

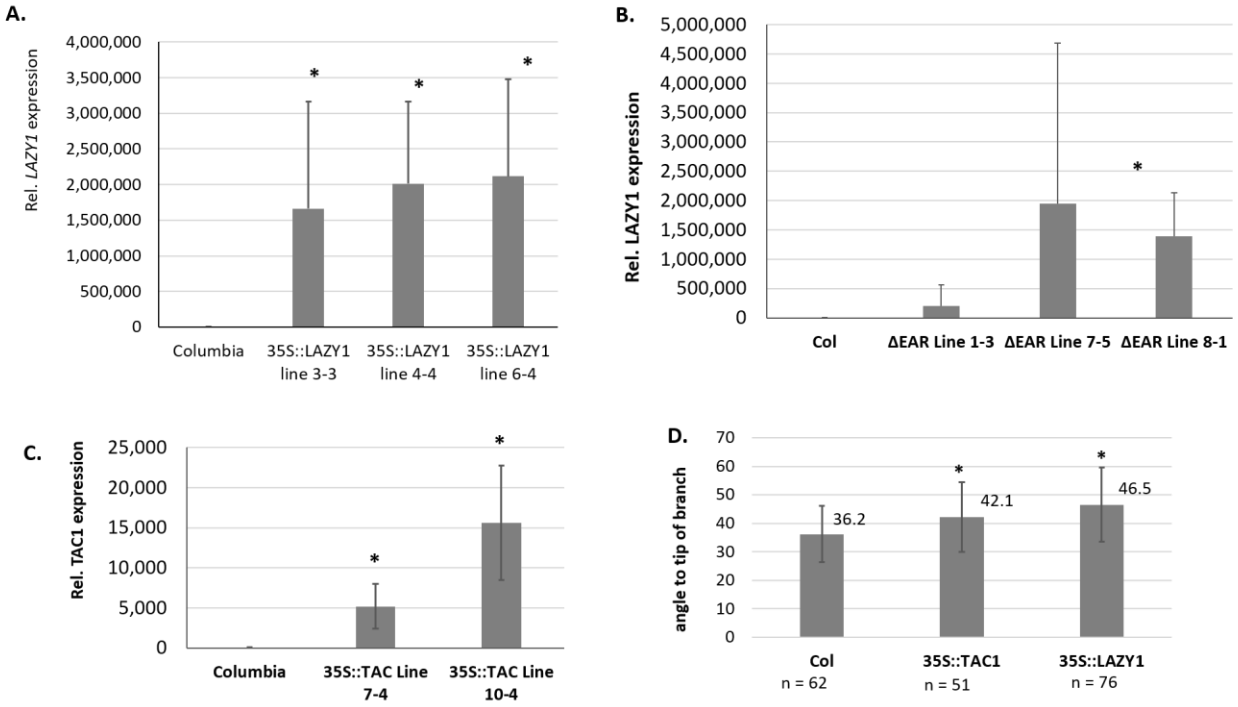

Supplementary Figure S4. 35S::TAC1 and 35S::LAZY1 arabidopsis transgene expression and branch angle measurements. (A-C) qPCR gene expression analysis for (A) 35S::LAZY , (B) 35S::LAZY delta EAR, and (C) 35S::TAC1 plant lines. Bars represent Standard Deviation of averages from between three and six biological replicates, each with three technical replicates. (D) Branch angle measurements for Columbia, 35S::TAC1 and 35S::LAZY1 plants, determined by measuring from the tip of the branch, just below the inflorescence, to the branch node and then up the stem to the node above. The number of angles measured is indicated below. For A-D, * indicates significant difference compared to Col ($p < 0.05$).

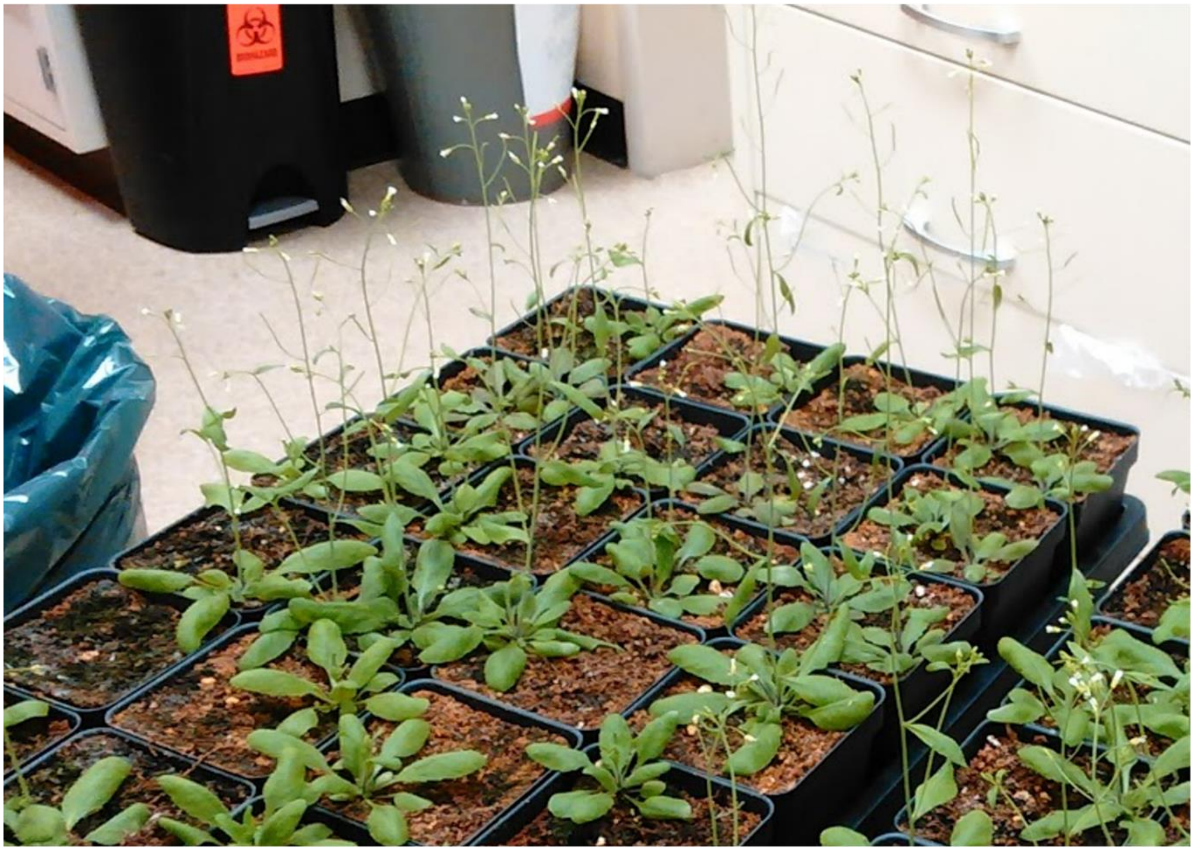

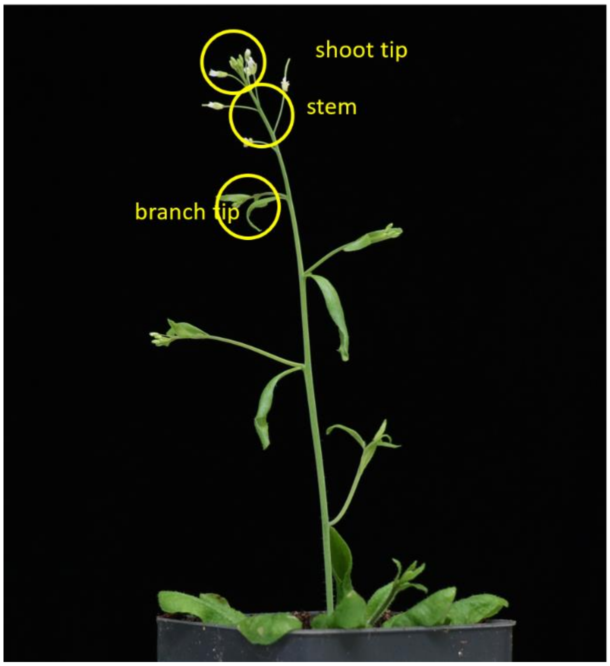

Supplementary Figure S5. Arabidopsis plants used for hormone analyses and tissues collection. The specific tissues harvested are indicated by yellow circles and text in bottom panel. Note: Late stage flower buds and open and senescing flowers were removed from shoot and branch tips when harvesting.

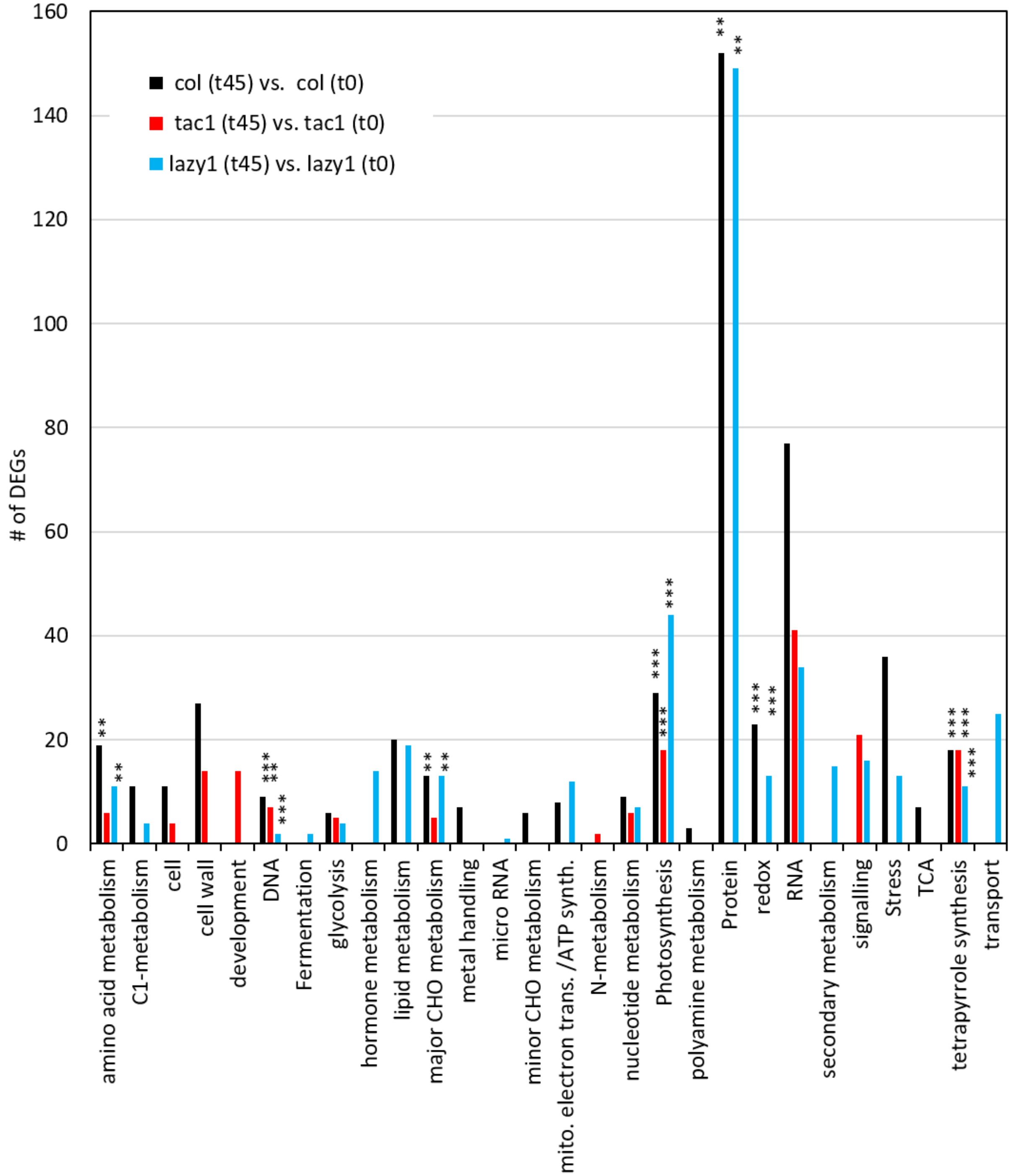

Supplementary Figure S6. Numbers of DEGs in the enriched MapMan categories from gravistimulated *tac1*, *lazy1* and columbia plants. Significance of each category is at least p <.05 but ** indicates p < 0.0001 (10e-5) and *** indicates p< 10e-10.

Table S1. Average shoot tip angles 30 and 60 minutes after 90-degree reorientation. The p-value is from a t-test between mutant and Columbia wild type plants

Timepoint		Col	*tac1*	*lazy1*	*tac1;lazy1*
30 min	30 min. avg tip angle	**178.9**	**179.1**	**184.0**	**186.7**
	Stdev	9.0	13.5	9.6	9.9
	SEM	1.6	2.7	1.8	2.0
	p=value		0.935393	0.035347	0.003366
60 min	60 min	**156.8**	**153.8**	**167.2**	**169.7**
	Stdev	14.1	17.5	15.5	12.7
	SEM	2.5	3.4	2.9	2.6
	p=value		0.487053	0.008011	0.000908

Supplementary Table S2. LC/MS/MS parameters used for hormone detection

	Mass number for MRM detection		Ionization and collision parameters (V)		
	**Q1**	**Q2**	**Retention Time**	**Cone Voltage**	**Collision Energy**
**SA**	137	93	2.43	28	16
**ABA**	263.1	153.1	2.75	22	10
**JA**	209.1	59	2.98	28	16
**IAA**	176.1	130.1	2.34	20	15
**IAA-ALA**	247.13	130.1	2.23	22	22
**IAA-PHE**	323.23	130.06	3.06	28	22
**IAA-IEU**	289.26	130.06	3.04	22	22
**[D_6]-ABA**	269.1	159.1	2.71	22	11
**[D_7]-IAA**	182.98	136.07	2.34	22	16

Supplementary Table S3. Numbers of RNAseq raw, trimmed, and mapped reads.

RNAseq sample name	# of raw reads	# of Reads after trim	# of mapped reads
t0 Col A	13,520,991	13,406,400	12,952,114
t0 Col B	17,795,774	17,604,498	16,963,649
t0 Col C	20,319,695	20,098,675	19,385,180
t0 lazy A	14,803,115	14,673,484	14,191,130
t0 lazy B	20,517,452	20,391,149	19,610,033
t0 lazy 6B	19,005,320	18,920,571	18,316,396
t0 tac B	16,333,140	16,173,574	15,637,693
t0 tac C	19,195,574	18,993,945	18,343,898
t0 tac 3B	20,566,598	20,480,277	19,885,452
t45 col 13	24,239,134	24,137,415	23,445,786
t45 col 1B	21,440,305	21,346,804	20,721,204
t45 col 5	21,837,466	21,741,416	21,095,877
t45 col 6	23,277,175	23,180,103	22,506,013
t45 lazy 13	18,808,385	18,727,725	18,071,892
t45 lazy 4	20,090,020	20,011,404	19,402,553
t45 lazy 5	21,946,335	21,857,020	21,190,594
t45 lazy 6	21,908,844	21,817,241	20,968,353
t45 tac 13	20,084,218	20,006,837	19,466,051
t45 tac 14	22,075,525	21,989,659	21,361,020
t45 tac 5	17,461,498	17,386,021	16,871,554
t45 tac 6	18,411,712	18,333,463	17,828,936
